# Cardiovascular, psychiatric, and neurological phenomena seen in mad honey disease: A clinical case report

**DOI:** 10.1002/ccr3.1889

**Published:** 2018-10-22

**Authors:** Tirtha Man Shrestha, Gaurav Nepal, Yow Ka Shing, Laxmi Shrestha

**Affiliations:** ^1^ Department of General practice and Emergency Medicine Tribhuvan University Teaching Hospital Maharajgunj Kathmandu Nepal; ^2^ Maharajgunj Medical Campus Tribhuvan University Institute of Medicine Maharajgunj Kathmandu Nepal; ^3^ National University of Singapore Singapore City Singapore; ^4^ Shantipur Hospital Kathmandu Nepal

**Keywords:** grayanotoxin, hallucinations, heart block, mad honey, neuropathy, rhododendron

## Abstract

Mad Honey Disease is characterized by intoxication symptoms secondary to over‐ingestion of grayanotoxin found in honey produced from rhododendron nectar. Cardiovascular symptoms are common, but psychiatric and neurological phenomena are rarely seen in this condition. Our case details a patient who presented with florid symptomology in all three aforementioned areas.

## 1 INTRODUCTION

*Apis laboriosa*, the Himalayan giant honey bee is the world's largest honey bee home to the mountainous regions of Bhutan, the Chinese province of Yunnan, India, and Nepal.^1^ At altitudes between 2500 and 3200 m, these bees build their nests storing red honey harvested from the nectar of white rhododendrons found at similar altitudes.^2^ The red honey is prized for its purported medicinal value, intoxicating and relaxing qualities primarily attributed to the grayanotoxin present in rhododendron nectar.^3^, ^4^ The Gurung people in Nepal are especially known for their harvest and consumption of this type of honey, both for its medicinal and hallucinogenic properties.^4^

Grayanotoxin, also known as rhodotoxin, can be found in the leaves, twigs, flowers, and secondary products of plants belonging to the Ericaceae family.^5^ As a voltage‐gated sodium channel molecular toxin targeted at binding the group II receptor site,^6^ intoxication of the said substance usually manifests with parasympathetic overactivity characterized by dizziness, hypotension, and cardiac rhythm disorders (eg, sinus bradycardia and atrioventricular blockade).^7^, ^8^, ^9^ More uncommonly, there have been reported cases of syncope, myocardial infarction, asystole, diplopia, convulsions, and hepatotoxicity caused by mad honey poisoning.^10^, ^11^, ^12^, ^13^

In this report, we present the case of a 52‐year‐old woman from the indigenous Gurung community of Nepal who presented to the hospital with a chief complaint of shortness of breath and a burning sensation in her chest after ingestion of red honey. She subsequently developed visual hallucinations, a clumsy broad‐based gait, and numbness of the peripheries.

## 2 CASE REPORT

A 52‐year‐old illiterate woman from the Gurung community of Nepal presented to the Emergency Department (ED) of Tribhuvan University Teaching Hospital (TUTH) with a chief complaint of a sudden onset, progressively worsening shortness of breath, and burning sensation in the chest. The episode started one day prior to the ED visit, immediately preceded by the consumption of four tablespoons of wild red honey. This was associated with visual hallucinations, blurring of vision, lightheadedness, a clumsy broad‐based gait, and numbness in the peripheries that lasted 3‐4 hours post‐honey ingestion. There was otherwise no fever and localizing signs of infection, no history of lower limb swelling, pleuritic chest pain, cough or sputum production, no gastrointestinal symptoms such as abdominal pain, water brash, nausea, vomiting, or loose stool, and the patient did not give any history of vertigo, confusion, or syncope.

The patient reported the visual hallucinations as a one‐episode sighting of a female god and wild beast at her home which no one else claimed to see. It resolved after sleeping. There was no associated auditory, tactile or gustatory hallucinations, and no associated passivity experiences, delusions, thought insertion or withdrawal. During the episode, the family members who were at the scene said the patient was muttering incomprehensibly, perhaps under the influence of her hallucinations.

She has no past medical or psychiatric history of note and no history of similar episodes in the past. She is not on any long‐term medications, and other than the honey, she did not take any new food, medication, traditional therapies, or supplements in the past month. She is a smoker of 25‐pack years and does not consume alcohol. She reported that a similar episode happened to her daughter few weeks back. Her daughter was feeling weak, given red honey and milk, and subsequently developed a cough, shortness of breath, and numbness in the peripheries. The incident resolved spontaneously and was otherwise not associated with any psychiatric symptoms.

On examination, the patient was ill‐looking but was oriented to time, person, and place. Her sphygmomanometric blood pressure read 60/40 mm Hg, her heart rate was 40 beats/min, her respiratory rate was 20 breaths/min, and her spO2 was 85% on room air. She was afebrile with a temperature of 98°F.

Neurological examination revealed 15/15 Glasgow Coma Scale, pupils that were equal and reactive to light, all cranial nerves intact with no focal neurological deficits of the limbs. Her gait was normal, and there was no nystagmus, dysmetria or dysdiadochokinesia. Cardiovascular and abdominal examination was unremarkable. There was however, decreased air entry in the bilateral lung bases and pitting edema on bilateral lower limbs, up to the level of the ankles.

Mental state examination revealed that the patient struck good rapport with no abnormal deviations in rate, rhythm, and quantity of speech. Although she was in acute discomfort, she largely had a positive affect and had no recent alteration in her mood. There were no obsessions, delusions, passivity experiences, illusions, or hallucinations identified other than the one episode she experienced just after ingestion of red honey. She demonstrated good insight into her illness and practiced good social judgment.

Baseline investigations were within normal limit. A 12‐lead electrocardiogram revealed sinus bradycardia with a first‐degree atrioventricular block. She was given supplemental oxygen (nasal prongs 4 L/min) and two doses of IV 0.6 mg atropine given 5 minutes apart. Her symptoms resolved rapidly over few hours and she subsequently had an uneventful discharge.

## 3 DISCUSSION

Grayanotoxins are a family of nitrogen‐free polyhydroxy cyclic hydrocarbons known to be present in honey produced from the nectar of rhododendron flowers.^6^ The indigenous honey hunters hold a belief that consuming it reduces the risk of heart disease, helps palliate the symptoms of gastrointestinal diseases and acts as a sexual stimulant.^14^ Over‐ingestion, however, may lead to toxic effects.

The commonly reported cardiovascular effects of over‐ingestion include hypotension, bradycardia (commonly secondary to sinus bradycardia, nodal rhythm, and complete atrioventricular block), and myocardial infarction. Previous case reports from Nepal and Turkey describe the fact that many of these cardiovascular manifestations are highly responsive to the administration of IV atropine.^7^, ^15^ This was similar in our case report.

Psychiatric effects, especially in the form of hallucinations, are also known to be linked to mad honey over‐ingestion. Intriguingly, these manifestations tend to precede the cardiac symptoms. In a case report featuring a woman aged 54 who ingested a quarter of a pound of honey,^16^ her cardiac symptoms of pallor and an irregular pulse came only after the onset of an episode of visual hallucinations and a burning sensation on her forehead. She later lost consciousness and developed generalized tonic‐clonic seizures, and was managed via the administration of an emetic and hypodermic injection of stimulants.

Interestingly, our patient also complained of neurological phenomena such as blurring of vision, a clumsy broad‐based gait, and numbness of the peripheries. A case series about grayanotoxin poisoning from Nepal previously reported honey‐intoxicated patients presenting with symptoms of blurring of vision, diplopia, nausea, and vomiting.^17^ However, an ataxic gait and numbness of the peripheries in the setting of mad honey intoxication, to our understanding, has not been reported previously in the literature.

Honey can be potentially therapeutic or toxic depending on its dose. Though various animal studies have been conducted to elucidate the mechanism by which grayanotoxins act, the dose‐response relationship in both animal and human systems has not been well established.^6^, ^10^ Lower doses of honey could have potentially therapeutic short‐term antiarrhythmic and long‐term cardiovascular benefits. As the old adage goes, “One man's poison is another man's cure”. More research is definitely warranted in this area to throw light on the therapeutic and toxic dose‐dependent properties of honey.

Apart from honey, grayanotoxins might be present in other products prepared from rhododendron. Further studies using liquid chromatography—tandem mass spectrometry can be done for determination of grayanotoxins in flower juice, wine, or boiled extract of rhododendron.

## CONFLICT OF INTEREST

The authors declare no conflict of interests in association with this manuscript.

## AUTHOR CONTRIBUTION

TMS: managed the patient and designed the study. GN and YKS: wrote the initial draft of the manuscript. GN, YKS, TMS, and LS: edited the draft and reshaped into this manuscript. All authors approved the final version of the manuscript and agree to be accountable for all aspects of the work in ensuring that questions related to the accuracy or integrity of any part of the work are appropriately investigated and resolved.
